# Supplementary material for: A meta-analysis of the value of circulating tumor cells in monitoring postoperative recurrence and metastasis of colorectal cancer
Source: PLoS One. 2022 Sep 19;17(9):e0274282. doi: 10.1371/journal.pone.0274282 (PMC9484659; doi:10.1371/journal.pone.0274282)
Supplement: Attachment — Submitted filename: Response to Reviewers.docx [file pone.0274282.s002.docx]

Response to Reviewers：

First of all, I would like to thank the editors and reviewers for taking the time to read my article and give professional opinions, and I will answer the questions raised below.

Major Comments:

1) In fact, I agree with the opinion of reviewer 1, and I wanted to do so at first. However, only two of the included papers, Nesteruk2014 and Yih-Huei2007, separate recurrence and metastasis, and the rest of the papers did not do so. Therefore, this study analyzed recurrence and metastasis together.
2) The flow chart has been uploaded again in the article.
3) I am very grateful for the opinions of the reviewers. At that time, the monitoring of ctDNA for postoperative recurrence and metastasis of colorectal cancer was not carefully found. In this study, the literature was reviewed to supplement the article.
4) The reason why the moderate monitoring value of CTC is obtained is mainly based on the AUC. The diagnostic value is moderate: AUC = 0.7～0.9. Our results suggest that in non-stage IV CRC, effectiveness of CTC in monitoring recurrence and metastasis can only be calculated medium. Therefore, in clinical practice, the specific location of postoperative recurrence or metastasis of CRC still needs to be used according to the specific clinical practice. In addition, there was a significant correlation between the dynamic changes of CTCs detection and imaging results.it was better than imaging examination in terms of timeliness, especially in metastatic CRC, CTCs detection could find clues of tumor metastasis earlier than imaging examination. Moreover, CTC combined with CEA, CA125, CA19-9 and other tumor markers have higher sensitivity and specificity, and may have higher diagnostic value. Therefore, CTCs cannot be used alone for the clinical diagnosis of CRC, nor can it replace other tumor markers and imaging examinations, but based on the results of CTCs detection, the frequency of these examinations can be reduced, or the time of these examinations can be prolonged, or combined with other tumor markers together with the detection to improve accuracy, the combination of CTCs detection and imaging can provide more clinical information. It can be used as one of the simple, effective and non-invasive diagnostic methods.

Minor Comments:

1) I'm sorry that B-ultrasound didn't make it clear, in fact it refers to Ultrasonography, the article has been revised.
2) CTC detection time point is also very important, but so far, the best detection time of CTCs for recurrence and metastasis after CRC is still unclear. Considering the number of included studies and clinical reality, we divided within one week after the operation and one week after the operation into two time points for subgroup analysis.
3) Sorry, there will be some grammatical errors in it as my native language is not English, I have fixed it.
4) In the included literature, there is no article that clearly suggests that EpCAM is used as a marker, so this article does not mention it.
5) Yes, the reviewer's opinion is correct, I have revised it.
