# Supplementary material for: A meta-analysis of the value of circulating tumor cells in monitoring postoperative recurrence and metastasis of colorectal cancer
Source: PLoS One. 2022 Sep 19;17(9):e0274282. doi: 10.1371/journal.pone.0274282 (PMC9484659; doi:10.1371/journal.pone.0274282)
Supplement: Attachment — Submitted filename: Response to Reviewers.docx [file pone.0274282.s003.docx]

Response to Reviewers：

First of all, I would like to thank the editors and reviewers for taking the time to read my article and provide valuable comments, from which I have benefited a lot. Next, please allow me to answer the questions of the editors and reviewers.

(1) I have checked the references in the article, there should be no retractions, I cite the literature strictly according to the requirements of your journal.
(2) Regarding the opinions of the reviewer, I have added them in the discussion part of the article. The red part of the article is written according to the opinions of the reviewer.
